# Role of Kinematics Assessment and Multimodal Sensorimotor Training for Motion Deficits in Breast Cancer Chemotherapy-Induced Polyneuropathy: A Perspective on Virtual Reality Avatars

**DOI:** 10.3389/fonc.2020.01419

**Published:** 2020-08-12

**Authors:** Cristian Axenie, Daria Kurz

**Affiliations:** ^1^Audi Konfuzius-Institut Ingolstadt Lab, Technische Hochschule Ingolstadt, Ingolstadt, Germany; ^2^Interdisziplinäres Brustzentrum, Helios Klinikum München West, Akademisches Lehrkrankenhaus der Ludwig-Maximilians Universität München, Munich, Germany

**Keywords:** breast cancer, chemotherapy-induced peripheral neuropathy, virtual reality, machine learning, sensorimotor rehabilitation, body sensors

## Abstract

Chemotherapy-induced polyneuropathy (CIPN), one of the most severe and incapacitating side effects of chemotherapeutic drugs, is a serious concern in breast cancer therapy leading to dose diminution, delay, or cessation. The reversibility of CIPN is of increasing importance since active chemotherapies prolong survival. Clinical assessment tools show that patients experiencing sensorimotor CIPN symptoms not only do they have to cope with loss in autonomy and life quality, but CIPN has become a key restricting factor in treatment. CIPN incidence poses a clinical challenge and has lacked established and efficient therapeutic options up to now. Complementary, non-opioid therapies are sought for both prevention and management of CIPN. In this perspective, we explore the potential that digital interventions have for sensorimotor CIPN rehabilitation in breast cancer patients. Our primary goal is to emphasize the benefits and impact that Virtual Reality (VR) avatars and Machine Learning have in combination in a digital intervention aiming at (1) assessing the complete kinematics of deficits through learning underlying patient sensorimotor parameters, and (2) parameterize a multimodal VR simulation to drive personalized deficit compensation. We support our perspective by evaluating sensorimotor effects of chemotherapy, the metrics to assess sensorimotor deficits, and relevant clinical studies. We subsequently analyse the neurological substrate of VR sensorimotor rehabilitation, with multisensory integration acting as a key element. Finally, we propose a closed-loop patient-centered design recommendation for CIPN sensorimotor rehabilitation. Our aim is to provoke the scientific community toward the development and use of such digital interventions for more efficient and targeted rehabilitation.

## 1. Introduction

Breast cancer remains the most prevalent chronic disease in women, with a predicted 5 million new cases reported annually and among those with higher survival rates (i.e., 5-years median survival in most developed countries is more than 80%). Following the initial diagnosis, the increasing number of survivors for relatively long periods highlights the need for adequate supporting rehabilitation interventions. Chemotherapy-induced polyneuropathy(CIPN) is among the most severe side effects of many regularly used chemotherapy drugs with a direct impact on the autonomy and quality of life of patients (1–3) with incidence varying between 12 and 96% in the case of taxane- and platinum-based chemotherapy (4–6). In both the prevention and treatment of CIPN, innovative rehabilitation approaches that can be implemented as an alternative to traditional pharmacological treatments are absolutely necessary (7). The current landscape of solutions is broad and diverse comprising:

**Table:** 

**Intervention**	**Study**
Wearable sensors	(8)
Sensorimotor balance training	(9)
Specialized physical exercise training	(10–13)
Physiotherapy	(14, 15)
Joint stabilizers and orthotics	(16)
Impact and vibration training	(17, 18)
Closed kinematic chain exercise	(19)
Visual computer-feedback balance training	(20, 21)
Transcutaneous electrical stimulation	(22)

These studies have laid the foundation for using technology in assessing, monitoring and controlling sensorimotor deficits associated with CIPN. Yet, none of them considered whole-body kinematic assessment of deficits and closed-loop deficit compensation. Our perspective sheds the light on the possibility to use commodity digital technologies, such as Virtual Reality (VR) and Machine Learning (ML), in combination, for personalized CIPN sensorimotor rehabilitation. We discuss the applicability of such a digital intervention facilitating motor deficits (e.g., gait and posture) in breast cancer patients.

We start our perspective by evaluating the impact chemotherapy has upon patient's sensorimotor system. We briefly introduce the typical metrics in sensorimotor deficits assessment and motivate the need kinematics assessment. Afterwards, we analyse the rehabilitation facilitating effects that VR offers, highlighting aspects, such as validity, multisensory integration, and neuroplasticity. Finally, we consolidate this thesis and propose a patient-centered design recommendation for CIPN sensorimotor rehabilitation.

## 2. Analysis of Sensorimotor Effects of CIPN

CIPN defines the harm to the peripheral nervous system experienced by a patient who has been administered a neurotoxic chemotherapeutic agent. The substances that most frequently cause CIPN in breast cancer are: vinca alkaloids, taxanes, and platin derivates.

### 2.1. Sensorimotor Deficit Assessment

Independent of the mechanisms of action, the targeted impact of such agents is on axonal transmission (1, 23, 24) with consequences leading up to neuronal apoptosis (25). CIPN generates sensory (26) as well as motor symptoms (27), with a high prevalence between 30 and 83% of the patients reporting persistent neuropathy, with 68.1% in <30 days following termination of the chemotherapy (6). Considering wider timescales, the average occurrence of CIPN was shown to be up to 28.7% in the first year after diagnosis, with more than 80% of the patients presenting symptoms after 6 months (28).

Joint deficits and dysfunction in the sensorimotor domain due to neurotoxicity can influence everyday activities, including gait, posture, and induce falls (29, 30). Any such deterioration in the sensory and motor information available can affect cortical integration of sensory and motor streams and the learning of internal models (31–33). Thus, any contradiction or inconsistency in sensorimotor input, or a decline in the reliability of perceptual information, facilitates the formation of sensorimotor aberrations, further hindering motor planning and execution during biomechanical processes, such as gait and postural control (34, 35).

In rehabilitation, the severity of such sensorimotor effects induced by CIPN is traditionally measured with subjective scales, such as the Functional Assessment of Cancer Therapy/Gynecologic Oncology Group Neurotoxicity (FACT/GOG-Ntx) and the European Organization for Research and Treatment in Cancer Quality of Life Quest CIPN 20/30 (EORTC QLQ—CIPN 20/30) (36). Their ability to ascertain deficiencies in body structure/function and restrictions in tasks during the therapy is confined and tied to the subjectivity of the patient (37). Typically, to support rehabilitation, such scales are complemented by quantitative scales of neurotoxic impact on perception [e.g., Fullerton Advanced Balance Scale (FABS), the Balance Evaluation Systems Test (BESTest)] (38). These standardized measures enrich whole-body assessment of sensorimotor deficits and provide patient motion context information (39), but lack the kinematic assessment. This is crucial, especially in the light of closed-loop training, where perceptual information needs to drive compensation of the deficits. In the next section, we analyse existing sensorimotor rehabilitation techniques used in CIPN with respect to kinematics assessment and sensorimotor training.

### 2.2. Sensorimotor Rehabilitation

Continuous monitoring after initiation of a neurotoxic intervention is critical for the formation and advancement of CIPN related symptoms (40). A large number of interventions have been developed to cope with the prevention or management of CIPN. With up to 26 identified complementary therapies for the typical neurotoxic agents used in typical chemotherapy schemes (i.e., Oxaliplatin, Cisplatin, Platinum/Taxane combination, Taxanes), Kalisch et al. emphasized the rather scarce space of efficient solutions in supportive therapies (7). We now analyse the most relevant interventions, highlighting the need for kinematics assessment in sensorimotor training.

#### 2.2.1. Primary Rehabilitation Strategies

Physical training can attenuate CIPN symptoms and reduce deficits manifested in both sensory and motor domains. Streckmann et al. supports such hypothesis by evaluating the adaptations of the neuromuscular system through EORCT-QLQ-CIPN20, FACT/COG-Ntx and a batch of sensorimotor exercises (41). Moreover, McCrary et al. (10) argued about the importance and the impact of multimodal exercise interventions on CIPN symptoms by emphasizing that neuromuscular system structural changes are sophisticated, and depend on the amount and vigor of the exercise. Interestingly, most relevant studies employing physical training (10–13) only evaluate a subset of motion parameters and pay the price for the high variability between interventions, such as the variety and length of exercises. In addition, the measurement procedures of the investigations were not compatible; for example, balance control was measured with different scales (9).

Providing a richer depiction of patient's motion parameters, the next level in CIPN sensorimotor rehabilitation focuses on the use of wearable sensors in physical training (8, 17, 19). Such an approach is an inexpensive, robust, and efficient method to screen motor performance deterioration by assessing spatio-temporal parameters of gait and balance [as shown by (8, 18)]. Extending the range of wearable rehabilitation modalities, whole-body vibration (WBV) training, has proven to attenuate sensorimotor deficits by de-conditioning skeletal muscles in order to reduce fall frequency (9). Our perspective is that such sensor-based rehabilitation systems have the potential to capture patient motion peculiarities, mainly in an open-loop configuration, but without compensating for deficits thorough closed-loop sensory feedback control (42).

#### 2.2.2. Advanced Rehabilitation Strategies

Targeting a closed-loop approach, the most advanced rehabilitation systems to date look at interactive motor adaptation training programs based on wearable sensors and visual animation (18). Such approaches exploit the benefit of the interactive joint movement feedback (43) and tap into error-dependent cortical learning rules between reference/desired motor action and the measured/executed motor action. Such techniques trigger adaptation of the neural internal model through closed-loop stimulation (32). Such systems promote the role of interactivity for motor rehabilitation through significant reduction of deficits (e.g., center of mass sway, disturbed postural stability, and coordination) (21). As vision dominates perception, visual computer-feedback balance training systems have the potential to improve balance in patients with CIPN (20), as measured by static-dynamic posture and balance scales and kinematic chains (19). Such rehabilitation methods emphasize the role of precise kinematic assessment and the potential that wearable sensors and interactive visual feedback have in compensating deficits, such as impaired joint proprioception (21, 44, 45).

## 3. Analysis of Virtual Reality for Sensorimotor Rehabilitation

### 3.1. Virtual-Real Feedback Loop

VR enables a rich patient-centered and patient-tailored interaction with the virtual environment via tools, such as head mounted devices which require less set-up and effort than would be needed to train a patient for a rehabilitation routine in the real environment (46). Moreover, VR technology can be used to deliver rich and targeted stimulation to a patient's nervous system and thereby take advantage of the plasticity of the brain to promote learning and re-learning and, hence, support sensorimotor rehabilitation (47). Significantly important and relevant for the adoption of VR in rehabilitation is the assessment of the consistency and resemblance of responses within a virtual environment to those in the corresponding physical environment. Transferring such responses to the real world after training would trigger perceptual-motor adaptation and motor re-learning (46, 48, 49). Hence brain's adaptation to VR changing environment circumstances could be similar to those in the physical world (50). The validity of the sensorimotor stimulation in VR is crucial, to make sure that there is a consistent match between the real and the virtual world, especially when assessing deficits. Supporting our hypothesis, Mellet et al. proved, through fMRI, that the neural correlates of recalling patterns acquired when moving in VR were equivalent to those evoked when walking in a physical environment, while ensuring movement quality and parameters (51). In CIPN rehabilitation the goal is improving movement quality through deficit compensation and we hypothesize that a digital intervention should guarantee that movements made in VR are equivalent in spatial and temporal structure to those which have to be reacquired in the physical environment. But the nervous system's underlying redundancy might eventually restrict the degree of motor recuperation that can be attained (52).

### 3.2. Compensation Through Adaptive Multisensory Integration

Human perception of motion and bodily self involves multisensory integration, with vision playing an especially important role (53–58). The flexibility of VR allows for a deliberately vivid visual experience that can be “mismatched” from the bodily signals (57, 59). Such a context allows to investigate how far would the brain perform multimodal integration and solve sensorimotor inconsistencies (60, 61). Such multisensory processes describe the impact that VR has upon the compensation of sensorimotor deficits, by guiding patients' perception toward correcting for the inconsistencies that the real deficits determine. Information from afferents in joints, muscles, and tendons as well as visual, vestibular, and auditory signals reach cortical integration areas in the frontal, parietal, and temporal lobes, where the fusion of these body signals occurs (55, 62–65). Thus, it appears likely that the self-identification of body motion and underlying kinematics is achieved in a similar manner, by dynamic multisensory integration processes known to be elicited in VR. After VR immersion, the brain is biased to estimate self-motion from visual cues in a vestibular cue conflicting process (66, 67). Our perspective is that such error correction mechanism, at cortical level, or the underlying re-cueing needs to occur in order to decorrelate the current percept from the adapted stimuli in VR. Thus, a subsequent re-weighting of sensory signals from the different modalities is necessary for optimal sensory integration (68, 69). Our hypothesis is that the stimulation environment richness modulates multisensory integration to compensate for sensorimotor deficits. Moreover, we believe that a digital intervention tapping into motion deficits should target the underlying correlates of cortical multisensory integration. VR has the potential to offer such targeted multimodal stimulation and elicit re-weighting and adaptation processes driving compensation. In order to drive compensation self-presence is vital. In VR self-presence is the feeling that my avatar is me. Using an interactive virtual environment that requires the use of avatars adds the component of self-presence, which turns the intervention into a personal experience (70) given the many factors that determine the cognitive connection or sense of identification between user and avatar (71). Our belief is that the precise mapping of one's motion peculiarities, the quality of the multimodal perception, and the consistency and validity of the internal model predictions can support a successful intervention (72, 73).

## 4. Recommendation for a Patient-Centered Virtual Reality Intervention

In this final section, given the generous frame we already unfolded, we provide a concept VR system for CIPN sensorimotor rehabilitation. Our aim is to provoke the scientific community to develop and use digital technologies for efficient patient-centered rehabilitation interventions.

**Figure 1:**
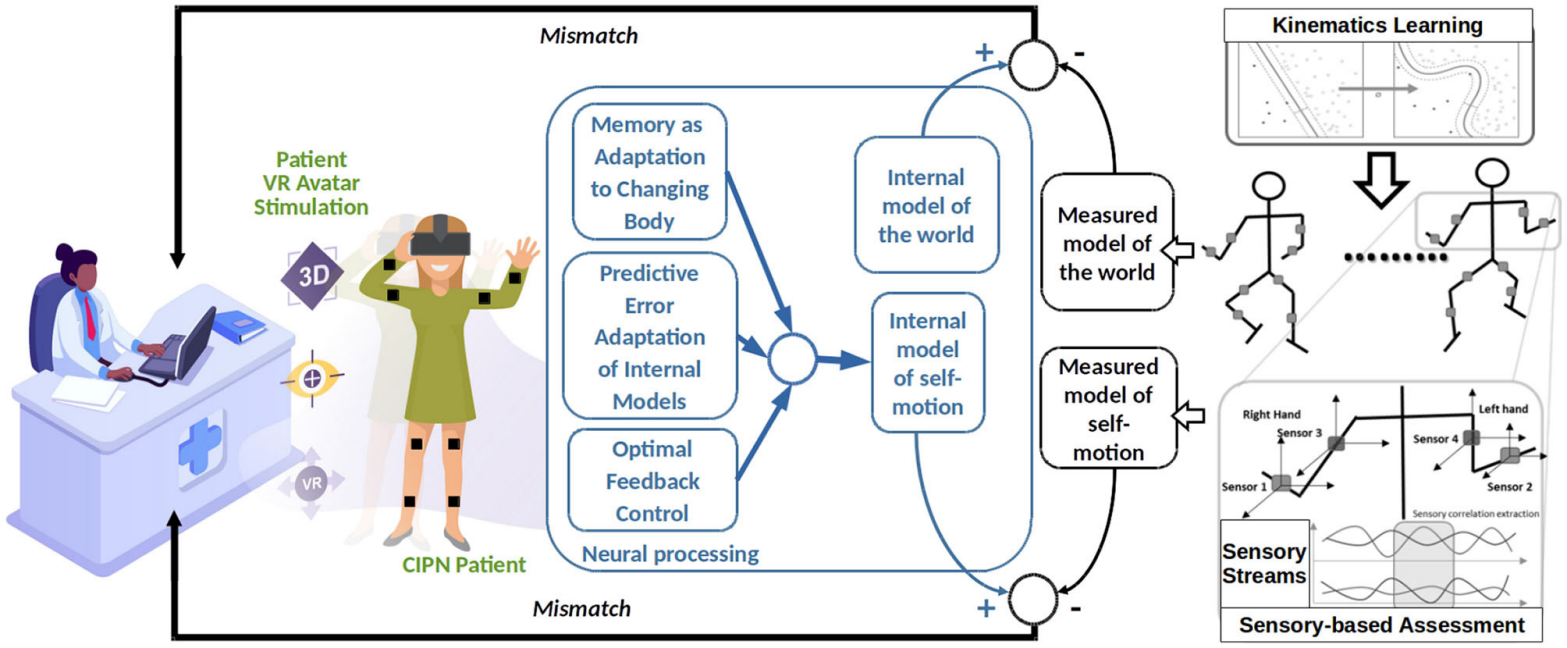
Recommended architecture for a patient-centered adaptive VR system for CIPN rehabilitation.

The conceptual architecture depicted in Figure 1 looks at kinematics assessment and multimodal sensorimotor training from a closed-loop control system perspective [as we previously shown in (74)]. Such a system would simultaneously update two models, a world model (i.e., avatar rendered in the training lab through VR system) and one self-motion model (i.e., obtained through wearable sensors). Such models can extract through machine learning algorithms the particularities of each patient's kinematics (i.e., joint angles and translation velocities ranges) and the world (i.e., peripersonal space and distal obstacles) using efficient correlation learning previously developed in Axenie et al. (75). Basically, by extracting the correlations among the various sensory streams describing the 3D motion of the patient (e.g., joint angles), the machine learning algorithms are able to regress the underlying dependencies in high-dimensional spaces among the motion variables (e.g., forearm w.r.t shoulder). The machine learning algorithms will, hence, use more complex features (e.g., trend detection, frequency, amplitude) and patterns underlying the sensory streams to extract motion peculiarities (e.g., drift, offset). The world model would hence describe the spatio-temporal aspects (i.e., patient executing a task: heading to an object and grasping), whereas the self-motion model would describe the kinematics (i.e., how to change trunk center of mass to move and arm joint angles to grasp). During the execution of the task each patient's brain will update the internal models of the world and the self (i.e., marked as neural processing in Figure 1). The mismatch between the internal neural models updates during the task and the external assessment of the rehabilitation system would then be used to parametrize the VR stimulation through the avatar. Preliminary experiments in Axenie et al. (76) confirm this hypothesis. The parametrization assumes the particular rendering of the patient avatar (i.e., with deficits) against a healthy patient avatar (i.e., no deficit). This way, the patient visually perceives the mismatch and will try to compensate for it.

Such an architecture has the potential to influence multisensory processes in the brain that account for both the perception of the surrounding space and self-motion. We hypothesize that this is possible through a series of processes that can also account as effects of VR stimulation, namely: the predictive adaptation of internal models, motion memory as adaptation to a changing body and optimal feedback control, respectively.

### 4.1. Predictive Error Adaptation of Internal Models

Under CIPN, peripheral nerves controlling muscles and tendons stiffness change, altering the kinematic and dynamic coupling between motor commands (e.g., torques) and motion of the limb (i.e., position and velocity). Along such progressive variations, the patient's body dynamics change. Hence in order to maintain a desired level of performance, the brain needs to be “robust” to such changes. This robustness may be attained through continuous update, or adaptation, of the internal kinematic model that predicts the sensory consequences of motor commands.

### 4.2. Memory as Adaptation to a Changing Body

Human brains exhibit patterns of learning and forgetting. Such processes unfold over different timescales where motion memory is consolidated as a consequence of the adaptation to a changing body. Chemotherapy neurotoxic effects, induces such changes in both sensory and motor systems. When considering sensorimotor rehabilitation, if a patient performs a task and observes an error, the brain tries to estimate the source of the error and to evaluate if the sensory encoding is still valid or exposed to aberrations.

### 4.3. Optimal Feedback Control

Our perspective is that under VR stimulation the patient's brain can act as a feedback compensator for sensorimotor deficits. This view taps into a core postulate of sensorimotor learning, namely optimal feedback control. In this framework, based on previously acquired experience, the brain computes a time-varying mapping from its internal states (i.e., internal models) into actions that minimize a total expected cost of responding to the incoming sensory input. Such learnt mappings/associations are essential to tune future motor responses to sensory information and, implicitly, compensate for eventual deficits.

## 5. Looking Ahead

Virtual environments facilitate the transfer and generalization of sensorimotor learning into the physical environment. Transfer is promoted if the avatar engagement with the virtual environments is equivalent to that expected in the physical world and the patient cognitive processing involved in task completion similar to the one required in the physical instantiation. Validating this hypothesis yields kinematics assessment and at the same time a rich multimodal stimulation.

Such a system could bring unique advantages through cost reduction for the clinics and an easy deployment for home-based rehabilitation. This offers an important advantage to the patients that will not need to visit the clinic often, rather focus on their daily lives allowing the system to non-intrusively monitor and learn their deficits and adaptively compensate for them. Our belief is that such a digital intervention holds the promise of offering truly personalized rehabilitation for life after cancer through a unique combination of technologies, such as VR and ML.

## Author Contributions

CA and DK contributed the conception and design of the perspective. CA wrote the first draft of the manuscript. DK wrote the sections of the manuscript. All authors contributed to the manuscript revision, read, and approved the submitted version.

## Conflict of Interest

The authors declare that the research was conducted in the absence of any commercial or financial relationships that could be construed as a potential conflict of interest.
